# Factors associated with immunosenescence during early adulthood in HIV-infected patients after durable efficient combination antiretroviral therapy

**DOI:** 10.1038/s41598-020-67100-8

**Published:** 2020-06-22

**Authors:** Eugenia Quiros-Roldan, Martina Properzi, Simone Paghera, Elena Raffetti, Francesco Castelli, Luisa Imberti

**Affiliations:** 1 Department of Infectious and Tropical Diseases, University of Brescia and ASST Spedali Civili Brescia, Brescia, Italy; 2 Centro di Ricerca Emato-oncologica AIL (CREA), Diagnostic Department, ASST Spedali Civili di Brescia, Brescia, Italy; 3 Department of Public Health Sciences, Karolinska Institutet, Stockholm, Sweden

**Keywords:** Immunology, Biomarkers, Diseases, Infectious diseases

## Abstract

Perinatally HIV-infected patients face the consequences of both chronic infection effects *per se* and long-term combination antiretroviral therapy (cART) on immunosenescence. Aims of our study were to evaluate which factors independently contribute to immunosenescence in HIV-infected young adults with a very different HIV infection duration (perinatally HIV-infected young individuals -pHIVy- and age-matched non perinatally HIV-infected youths –npHIVy), after durable  efficient cART. We considered low thymic and bone marrow output, respectively evaluated by quantifying T-cell receptor excision circles (TRECs), K-deleting recombination excision circles (KRECs), and shorter telomeres lenght (TL) as surrogate biomarkers of immunosenescence. Twenty-one pHIVy and 19 npHIVy (with a mean HIV duration of 3–8 years) were included; mean age was 27 years for both groups. Immunosenescence biomarkers were comparable between pHIVy and npHIVy (despite longer HIV-infection, higher frequency of AIDS events, past cART-free periods and concomitant chronic viral infections in pHIVy). At the multivariate analysis, CD4+ was the only variable independently associated with TRECs and TL. Our data suggest that a good level of thymic activity can compensate the deleterious effects of past periods without cART, if HIV replication is suppressed for a sufficient time.

## Introduction

In general population, aging has been related to chronic inflammation and modifications in innate and adaptive immunity, including decreased thymic output, increased levels of activation markers (CD38/HLA-DR), shift of naïve T cells towards memory cells with oligoclonal expansions and extensive proliferative history (represented by CD57 overexpression) and shorter telomeres^1^. These age-associated immune changes are generally referred to as “immunosenescence”. Chronic viral infections are considered major contributors towards inducing immunosenescence^2^, which persists over time during HIV infection, despite efficacious combination antiretroviral therapy (cART)^3^. HIV infection can induce thymic damage through thymocytes self-killing or disruption of the thymic stromal architecture, resulting in defective thymopoiesis and apoptosis of CD4+ T-cells, and in several alterations in T-cell compartment, most notably a shift from naïve to terminally differentiated cells^4^. Immune recovery may occur in some patients during cART; however, extensive thymic damage can hamper immune reconstitution^5,6^. Cytomegalovirus (CMV) infection has also a considerable effect on circulating T cells in healthy individuals and it has been implicated in immunological aging^7,8^. For instance, CMV-infected individuals show a more differentiated memory T-cell compartment, together with a decreased CD4+/CD8+ ratio, an expansion of CD4+ and CD8+ T cells lacking CD28 but expressing CD57 marker, and a reduction in telomere length (TL), indicating an increased T-cells proliferative history^7,9^. Chronic CMV/HIV coinfection has been associated with gradual deterioration of the immune system, with homeostatic changes and low CD4+ T-cell counts. This is particularly true for naïve T-cells, possibly due to thymic involution and decreased T-cells renewal capacity, leading to inadequate T-cell reconstitution^10^. Similar findings have been described in patients with chronic hepatitis C (HCV) infection^11^ and in individuals infected with common viral pathogens, e.g. influenza virus^12^.

Perinatally HIV-infected adults should face the consequences of both chronic infection effects *per se* and long-term cART on immunosenescence^3,13^. In fact, these patients are likely to have complex clinical histories, CMV and HCV coinfections, heavy antiretroviral drug exposure and multi-class drug resistant virus, as a consequence of low adherence to cART as well as high levels of HIV cumulative viremia^14^.

Most of the previously published studies about the role of HIV on the progressive decline of the immune system function, contributing to premature aging, demonstrated that while in infected adults, cART only partially reverses HIV-mediated immune defects^15,16^, in HIV-positive children therapy induces an early sustained increase in naïve CD4+ T cells, likely reflecting a greater thymic activity^17,18^. The reasons for these differences between children and adults are unclear: while one contributing factor may be the much higher thymic export of naïve T-cells in children than in adults, other significant immunological variations between individuals (possibly induced by age, sex, antigenic exposure and environmental factors) may also play a role^19^.

T-cell receptor excision circles (TRECs) and K-deleting recombination excision circles (KRECs) are circular excision products formed during genomic rearrangements of T-cell receptor (TR) and immunoglobulin loci; they are stable and not replicated during cell division^20^. As such, TRECs and KRECs are considered as indicators for thymic and bone marrow output and, indirectly, as surrogate biomarkers of a senescent phenotype. Recently, we described the differences on immunosenescence (comparing the numbers of TRECs, KRECs and TL levels) between HIV-positive (both long-living perinatally and non-perinatally infected on effective cART) and age-matched uninfected individuals^21^. We did not find any statistically significative difference in numbers of TRECs and KRECs in HIV-infected patients compared to healthy controls, although they showed statistically significant lower TL values and a restricted TR repertoire. However, we did not explore which clinical, epidemiological, virological and therapeutic characteristics may have an impact on immunological aging in these peculiar patients, considering the inhomogeneities of these factors and the individual variability in the numbers of TRECs, KRECs and TL levels. Thus, aim of the present study was to evaluate which factors independently contribute to immunosenescence in HIV-infected young adults, both perinatally HIV-infected young individuals (pHIVy) and age-matched non perinatally HIV-infected youths (npHIVy), after durable efficient cART.

## Methods

In this cross-sectional study we included all perinatally HIV-1-infected patients transferred from the Pediatric Unit to adult care at 18 years of age and in active follow-up at our adult Department for HIV infection at December 2018. Data on perinatal HIV infection were retrospective collected. Information included: demographic and clinical characteristics, HIV-1 related diseases and comorbidities, antiretroviral regimens duration and time free of cART. We also included a group of age-matched npHIVy with HIV infection duration >12 months (to exclude acute HIV infection). For pHIVy and npHIVy exclusion criteria were an ongoing HCV treatment or any serious concomitant disease/ongoing opportunistic infection. All patients were treated with cART according to current guidelines for HIV treatment.

The number of TRECs and KRECs was simultaneously quantified by duplex quantitative Real-Time PCR (qPCR), starting from DNA obtained from peripheral blood mononuclear cells, as previously reported^21^. Results were expressed as TRECs and KRECs/ml of blood. Similarly, TL was quantified as previously reported with minor modifications^21^ by measuring telomere (T) and beta-globin gene (S, single copy gene) signals in the same well, in comparison to a reference DNA, to yield relative T/S ratios proportional to average TL.

We used common statistical methods for mean and proportion comparisons as appropriate. We assessed the associations between variables using linear regression univariate and multivariate models.

This study was conducted according to the Declaration of Helsinki and to principles of Good Clinical Practice (GCP). Written informed consent was obtained from all subjects. The study protocol was approved by the local Ethic Committee of Brescia Province (Comitato Etico di Brescia - March 2018 NP 3061). Data were analyzed anonymously and each subject was identified using an alphanumerical code.

## Results

### Cohort characteristics

We included 21 pHIVy (7 males and 14 females) and a control group of 19 age-matched npHIVy (12 males and 7 females), sexually infected in the last 3–8 years prior to the enrollment in our study. Demographic and clinical characteristics of study population are summarized in Table 1. Mean age was 27 years for both groups (ranging from 22 to 35 years in pHIVy group and from 20 to 31 years in npHIVy group) at the time of study; remarkably, HIV duration was significantly different. Co-infection with CMV was present in 62% (n = 13) of pHIVy and in 36% (n = 7) of npHIVy. Almost half of pHIVy (n = 9; 42.8%) had a history of clinically significant immunodeficiency related to HIV-infection (AIDS-defining events; CDC class C definition) at some point of their follow-up (see Table 2). Two patients (patients #16 and #20) had three different diagnosis of AIDS events and two patients (#12 and #14) showed a total of two past AIDS-defining diseases. The most frequent AIDS events were: pulmonary *P. jiroveci* infection, disseminated CMV disease, progressive multifocal leukoencephalopathy (PML), recurrent bacterial pneumonia (defined as two episodes occurring within a 12-month period) and candidiasis of the oesophagus (n = 2 for any). CD4+ T cell nadir was not available for the pHIVy group. None of the npHIVy were CDC class B or C or showed any AIDS-defining illnesses, except one patient who experienced a disseminated CMV infection; their mean CD4+ nadir was 626.6 cell/µl (SD: 202.6). All participants were receiving an efficient cART at the time of study and their plasma viremia was <50 copies/ml. Mean duration of last period with HIV suppression before the enrollment in our study was 1583.9 + 1023.7 days for pHIVy and 1085.8 + 434 days for npHIVy. During their life pHIVy received a mean of 9 cART regimens, while npHIVy received a mean of 3 different therapies. For pHIVy, total mean time without cART since birth was 3836.3 days (SD: 2783.5). npHIVy after initiating their first cART regimen never spent time without therapy.

**Table 1:** Characteristics of included subjects at the enrollment (T1).

	pHIVy patients (n = 21)^*^	npHIVy patients (n = 19)^a^
Age (years); mean (range)	27 (22–35)	27 (20–31)
Males; mean (%)	7 (33)	12 (63)
Years with HIV; mean (range)	27* (22–35)	4.5* (3–8)
HIV viral load <50 copies/ml; n	21	19
Hepatitis B surface antigen positive; n (%)	1 (4.76)	1 (5.26)
Hepatitis C virus Ab positive; n (%)^c^	5 (23.81)	1 (5.26)
Cytomegalovirus IgG positive; n (%)	13 (61.90)	7 (36.84)
Toxoplasma gondii IgG positive; n (%)	4 (19.04)	2 (10.52)
cART regimens prescribed before T1; mean (range)	9 (2–16)	3 (1–5)
Mean duration of last period with HIV suppression before T1; days (SD)	1583.9 (1023.7)	1085.8 (434)
**Treatment prescribed at T1:**		
PI+ 2 NRTI; n (%)	4 (19)	5 (26)
NNRTI+ 2 NRTI; n (%)	3 (14)	8 (42)
INI+ 2 NRTI; n (%)	6 (29)	6 (32)
INI+ PI; n (%)	7 (33)	0
INI+ NNRTI; n (%)	1 (5)	0

n, number of patients; SD, standard deviation; PI, protease inhibitor; NRTI, nucleoside reverse transcriptase inhibitor; NNRTI, non nucleoside transcriptase inhibitor; INI, integrase inhibitor.

*P < 0.001 (P-value was calculated by unpaired t test for continuous variables).

^a^9 patients were heterosexuals, 8 homosexuals and 2 bisexuals.

^b^All reached a Sustained Virological Response (SVR) after treatment.

**Table 2:** Demographic and clinical characteristics of pHIVy (n = 21) at enrollment (T1).

Pts No.	Sex (M/F)	Age (years)	CD4+ cell/uL (%) at T1	CD8+ cell/uL (%) at T1	Total time without cART (days)*	Time with undetectable^§^ VL before T1 (days)	AIDS events (year)	No. cART lines	No. virological failures	CMV IgG (pos/neg)
#1	F	35	1150 (38.8)	912 (30.8)	1056	2853	PCP (1998)	9	3	pos
#2	F	24	803 (29.3)	1455 (53)	2195	2967		4	1	pos
#3	F	24	874 (40)	716 (32.7)	1437	3095		8	1	pos
#4	F	22	1052 (39)	1075 (39.9)	7	1346		7	1	pos
#5	M	23	864 (36.8)	614 (26.1)	2030	1029	dCMV (1995)	7	2	pos
#6^	F	22	193 (28.7)	244 (36.2)	670	1267		12	4	neg
#7^	F	24	1106 (30)	1734 (47.1)	851	381	EC (1996)	11	5	NA
#8^	F	26	1195 (33.8)	1204 (34.1)	1515	3073		12	5	pos
#9^	F	27	676 (38.7)	816 (46.7)	3607	477		12	5	NA
#10	F	31	693 (38.2)	886 (48.7)	5167	1313		14	4	pos
#11	F	28	526 (16)	1840 (55.9)	6753	722	RBP (2009)	12	2	pos
#12	F	29	339 (24.1)	777 (55.2)	6525	1866	ADC, PML (2013)	12	3	pos
#13^§^	M	27	855 (31.7)	1131 (39.8)	5388	3137		2	0	pos
#14	F	30	61 (5.7)	544 (51)	7721	269	PCP, EC (2018)	12	3	pos
#15	M	25	682 (25.6)	1363 (51.2)	7906	514	TB (1998)	2	1	NA
#16	F	28	85 (7.4)	721 (62.3)	1618	312	PML, EC, dCMV (2017)	15	7	pos
#17	M	28	491 (33.6)	713 (48.8)	6914	2909		3	0	NA
#18	M	30	878 (35.3)	1065 (42.8)	6959	783		7	1	NA
#19^	M	26	1427 (48.5)	850 (28.9)	7424	1553		3	0	pos
#20	M	24	536 (36.2)	362 (24.5)	1029	1140	MAC, NHL, RBP (2004)	16	7	neg
#21	F	28	1071 (50.4)	567 (26.7)	3791	2255		3	0	neg

Pts, patients; M, male; F, female; cART, combination antiretroviral therapy; pos, positive; neg, negative; PCP, Pneumocystis pneumonia; dCMV, disseminated Cytomegalovirus infection; EC, esophageal candidiasis; PBR, recurrent bacterial pneumonia; ADC, AIDS dementia complex; PML, progressive multifocal leukoencephalopathy; TB, Tuberculosis; MIC, Mycobacterium avium complex infection; NHL, non-Hodgkin lymphoma; NA, unavailable data.

*calculated from birth.

^§^HBsAg positive patient.

^^^HCVAb positive patient. All coinfected patients were successfully treated with Directly Acting Antivirals (DAA).

^§^defined as HIVRNA <50 copies/mL.

### Immunological parameters

No differences between pHIVy and npHIVy were observed for CD4+ cells (absolute number or percentage), CD4+/CD8+ ratio, number of TRECs and KRECs or TL levels. Only CD8+ percentage was significantly higher in pHIVy than in npHIVy (42% vs 33.9%, p = 0.015, see Table 3); CD4+/CD8+ ratio was lower but not statistically significant (0.87 vs 1.16, p = 0.057). We evaluated the relationship between demographics and clinical characteristics, including HIV duration, and proxies of immunosenescence, including low thymic function (TRECs), bone marrow output (KRECs) and short TL (T/S), using univariate linear regression models (Table 4). CD4+ cell quartile increase (coef 4882.10; 95% confidence interval (CI) 2061.7; 7702.5) and CD4+/CD8+ quartile (coef 3174.0; 95% CI 249.6; 6098.3) were associated with the number of TRECs. CD4+ quartile increase (coef 0.14; 95% CI 0.01; 0.2) and CD4+/CD8+ quartile (coef 0.11; 95% CI 0.03; 0.2) were also associated with TL, while no association was found for any variables and the number of KRECs. No differences were found in immunesencescence proxies in subjects with and without CMV infection. Among pHIVy we evaluated the relationship between the risk factors for HIV acquisition and immunosenescence outcomes. Subjects infected through heterosexual intercourses had higher levels of TRECs compared to men who have sex with men (coef 8129.92; 95% CI 1405.62; 17665.5). The suppression time was also not associated with any immunosenescence outcome (data not shown).

**Table 3:** Immunological parameters of included subjects.

	pHIVy patients mean (range)	npHIVy patients mean (range)	P value^a^
CD4+/µl	740	849	0.319
	(61–1427)	(292–1525)	
CD4 + %	31.8	37.3	0.075
	(5.7–50.4)	(26.1–47.8)	
CD8+/µl	932	758	0.124
	(244–1840)	(470–1224)	
CD8 + %	42	33.9	0.015
	(24.5–62.3)	(24.5–50.7)	
CD4+/CD8+ ratio	0.87	1.16	0.057
	(0.1–1.9)	(0.6–1.9)	
TRECs/ml	16548	15674	0.808
	(1178–50280)	(3065–45188)	
KRECs/ml	24598	20189	0.469
	(2731–73456)	(3108–61869)	
TL^b^	1.01	0.98	0.708
	(0.53–1.38)	(0.54–1.46)	

pHIVy, perinatally HIV-infected youths; npHIVy, non perinatally HIV-infected youths; TRECs, T-cell receptor excision circles; KRECs, K-deleting recombination excision circles; TL, telomeres length.

^a^P-values were calculated for all variables by unpaired t test. P < 0.05 were considered significant.

^b^TLs were expressed as T/S ratio.

**Table 4:** Univariate linear regression analysis.

	TRECs		KRECs		TL	
	Regression coefficient (CI)	p	Regression coefficient (CI)	p	Regression coefficient (CI)	p
Age (years)	−402.2 (−1593.0;789.5)	0.499	61.1 (−1970.6;2092.7)	0.952	−0.02 (−0.5;0.01)	0.112
Gender	−2146.3 (−9375.4;5082.9)	0.551	−6061.0 (−18206.6;6084.7)	0.319	−0.06 (−0.2;0.1)	0.483
Nationality	−4463.5 (−12710.8;3783.9)	0.28	−3661.8 (−17804.6;10481.1)	0.603	0.02 (−0.2;0.2)	0.817
CD4+/µl	17.9 (8.8;27.0)	<0.001			0.0005 (0.0002;0.0007)	<0.001
CD4+ quartile increase	4882.10 (2061.7;7702.5)	0.001			0.14 (0.01;0.2)	<0.001
CD8+/µl	9.2 (−0.4;19.0)	0.059			0.00002 (−0.0002;0.0003)	0.904
CD8+ quartile increase	2341.6 (−664.0;5347.2)	0.123			0.02 (−0.1;0.1)	0.6
CD4+/CD8+ ratio	9034.0 (2116.2;15951.9)	0.012			0.31 (0.2;0.5)	<0.001
CD4+/CD8+ ratio quartile	3174.0 (249.6;6098.3)	0.034			0.11 (0.03;0.2)	0.003
Years with HIV	28.7 (−348.2;405.5)	0.879	154.9 (−481.8;791.6)	0.625	−0.003 (−0.01;0.01)	0.438
HBsAg+	−4931.4 (−21495.08;11632.1)	0.55	−16185.3 (−43880.3;11509.7)	0.244	0.13 (−0.3;0.5)	0.517
HCVAb+	3678.6 (−6407.4;13764.7)	0.465	8626.1 (−8351.6;25604.1)	0.31	0.09 (−0.2;0.3)	0.465
CMV IgG+	10366.1 (−7943.9;28676.2)	0.252	14491.3 (−16793.0;45775.7)	0.346	−0.05 (−0.5;0.4)	0.811
T. gondii IgG+	−2779.8 (−12322.2;6762.6)	0.557	7475.4 (−7935.6;22886.4)	0.331	−0.09 (−0.3;0.1)	0.4
No. of AIDS diagnosis	−2069.8 (−10418.8;6279.1)	0.619	12335.5 (−1268.1;25939.0)	0.074	−0.16 (−0.4;0.04)	0.108
NNRTI	−989.5 (−8474.7;6495.6)	0.79	684.8 (−12008.6;13378.3)	0.914	−0.09 (−0.3;0.08)	0.273
NRTI	−4150.2 (−12728.7;4428.3)	0.334	−12567.0 (−26695.0;1560.9)	0.08	0.05 (−0.2;0.3)	0.612
TDF	−8997.8 (−16340.6;−1654.9)	0.018	−3602.7 (−16962.4;9757.0)	0.588	−0.15 (−0.3;0.03)	0.092
TAF	−2451.9 (−9795.9;4892.2)	0.503	−12202.1 (−24261.1;−143.1)	0.047	−0.03 (−0.2;0.1)	0.741
INI	−197.1 (−7487.6;7093.5)	0.957	2123.7 (−10210.8;14458.2)	0.729	−0.018 (−0.2;0.2)	0.836
PI	3919.1 (−3229.3;11067.6)	0.274	10445.8 (−1374.2;22265.7)	0.082	0.04 (−0.1;0.2)	0.651

TRECs, T-cell receptor excision circles; KRECs, K-deleting recombination excision circles; TL, telomeres length (expressed as T/S ratio); CI, confidence interval; HBsAg, hepatitis B surface antigen; HCVAb, hepatitis C virus antibody; CMV, cytomegalovirus; NNRTI, non nucleoside transcriptase inhibitor; NRTI, nucleoside reverse transcriptase inhibitor; TDF, tenofovir disoproxil fumarate; TAF, tenofovir alafenamide fumarate; INI, integrase inhibitor; PI, protease inhibitor. CD4, 1st quartile (61.00–536.00), 2nd quartile (613.00–803.00), 3rd quartile (818.00–1052.00), 4th quartile (1071.00–1525.00); CD8, 1st quartile (244.00–631.00), 2nd quartile (643.00–777.00), 3rd quartile (790.00–1065.00), 4th quartile (1075.00–1840.00); CD4/CD8, 1st quartile (0.10–0.60), 2nd quartile (0.70–1.00), 3rd quartile (1.10.1.30), 4th quartile (1.40–1.90).

In adjusted models, the only factor associated with the number of TRECs and TL was CD4+, measured both as cell counts (data not shown) and quartile increase (coef. 4829.1; 95%CI 1988.7; 7669.5 and coef. 0.16; 95% CI 0.1; 0.2, respectively) (see Table 5). Similar associations were found considering CD4+/CD8+ ratio quartile instead of CD4+ and CD8+ in sensitivity analysis (data not shown).

**Table 5:** Multivariate regression analysis.

	TRECs		TL (T/S)	
	Regression coefficient (CI)	p	Regression coefficient (CI)	p
Age (years)	218.7 (−765.5;1202.8)	0.654	−0.01 (−0.03;0.01)	0.401
Gender	−1178.1 (−7365.8;5009.6)	0.7	−0.07 (−0.2;0.1)	0.3
Vertical transmission	3696.4 (−2561.3;9954.2)	0.237	0.10 (−0.04;0.3)	0.154
CD4+ quartile increase	4829.1 (1988.7;7669.5)	0.002	0.16 (0.1;0.2)	<0.001
CD8+ quartile increase	1024.3 (−1825.8;3874.4)	0.469	−0.03 (−0.1;0.03)	0.297
TDF	−5469.2 (−12450.3;1512.0)	0.12	−0.02 (−0.2;0.1)	0.773

TRECs, T-cell receptor excision circles; KRECs, K-deleting recombination excision circles; TL, telomeres length (expressed as T/S ratio); CI, confidence interval; TDF, tenofovir disoproxil fumarate.

## Discussion

In this peculiar group of pHIVy (virologically suppressed for several years despite lifelong HIV infection, with high frequency of past AIDS events, past long periods without cART and in the presence of widespread CMV infection) the number of TRECs and KRECs or TL levels were comparable to that of age-matched young adults with recent sexually acquired HIV-infection, although pHIVy showed higher CD8+ cells and lower CD4+/CD8+ ratio. In addition, we have previously showed that the values of TRECs and KRECs in the two groups of infected patients were comparable to those of age-matched uninfected individuals, although HIV-infected patients showed statistically significant lower TL values and a restricted TR repertoire compared to healthy controls^21^. Therefore, almost 30 years of HIV-infection and concomitant chronic viral infections did not apparently affect thymic and bone marrow release (or TL) in virosuppressed pHIVy. In our study, T-cell senescence markers (low TRECs and shorter TL in PBMCs) only correlated with recent CD4+ cell number.

Nowadays, it is generally accepted that HIV infection leads to both immunosenescence and inflamm-aging, as chronic inflammation induces an aged immune profile, even in individuals on cART and with undetectable viremia. However, combined effects of HIV infection and aging in pHIVy are not well defined. Our results complete those obtained on younger pHIVy. In fact, Blanche *et al*.^22^ showed that, after a mean of 17 years of HIV infection (meaning patients 10 years younger than ours), thymic activity (measured as CD4+ CD45RA+ CD31+ recent thymic emigrants or RTE) and naïve CD4+ cells number were conserved, while RTE positively correlated with CD4+ cells current number. Moreover, Aguilera-Sandoval *et al*.^18^ compared thymopoiesis of viremic and aviremic pHIVy to healthy individuals (all with mean age of 17 years). They did not find evidence of more robust thymopoiesis in pHIVy, as RTE and naïve subsets were relatively normal to elevated in the aviremic perinatally-infected group and slightly reduced in viremic pHIVy (however these values were not statistically different). More recently, Fastenackels *et al*.^23^ reported that pHIVy with uncontrolled HIV replication showed similar immune alterations to those of older HIV-infected patients with a decrease of progenitor cells (CD34+ CD45+), B lymphocytes (CD21+) and NK (CD56+/CD3−) cells and an increase of memory T cells (CD57+ CD8+).

These data, together with ours, suggest that a good level of thymic activity can compensate the deleterious effects of past periods without cART (with consequent viremic periods and previous AIDS clinical events) during childhood, if HIV replication is suppressed for a sufficient time. Here, an efficient cART seems to stabilize HIV immunosenescence in pHIVy at the same level of young adults with much shorter duration of HIV infection, at least until their third decade of life. In fact, in our study, markers of T-cell senescence (low TRECs and short TL) were similar in pHIVy and npHIVy and correlated only with CD4+ cells, regardless of HIV duration, coinfections or past AIDS events.

Concerning cART regimens, at the univariate analysis we found a negative association between tenofovir disoproxil fumarate (TDF) and the numbers of TRECs (not confirmed at the multivariate regression). Moreover, although not statistically significant, TL levels seemed to negatively correlate with TDF assumption. Previous studies underlined the possible role of nucleoside reverse transcriptase inhibitors (NRTIs) in TL levels shortening, as they inhibit telomerase activity *in-vitro*^24^. These evidences may suggest that HIV-related immunosenescence can also be ascribed to cART adverse effects, despite more studies are needed in order to better understand the complex mechanisms regulating aging in HIV-infected individuals.

Differences in thymic function in severely lymphopenic HIV-infected adults are associated with distinct immune recoveries that diverge soon after cART initiation^25^. It is well known that thymus serves as the central organ of immunologic self-/non-self-discrimination and, early in life, thymic export establishes the size and the diversity of T-cell pool. Indeed T cells released from thymus react with foreign antigens, but not with self-antigens. However, during bone marrow or solid organ transplantations appropriate donor antigen presentation in thymus induces tolerance to allografts^26^. Similarly, prenatal and neonatal periods may represent critical stages for immune development during which antigenic exposures can have long-term consequences for immune system shaping. Accordingly, it has been demonstrated that the developing immune system in prenatal period is fully functional, but it is highly suppressed because of the involvement of different types of regulatory cells (Treg)^27^. Tolerance provided by Treg is bidirectional and is necessary to maintain an immunosuppressive setting in order to defend both the mother and fetus in the antenatal period and the neonate after birth. In addition, early life exposures to antigenic stimulation are key determinants to shape the adult-like immune state^28^. Therefore, our data suggest that early-life exposure to HIV may have long-lasting consequences, with the adaption of immune system to the infection, but without thymic impairment. This should allow a normal production of new T-cell instead of a preferential peripheral proliferation of memory T cells, even if patients experienced viremic periods in the past.

The only differences found between pHIVy and npHIVy (ruling out HIV infection duration) regard CD8+ percentage and CD4+/CD8+ ratio. Recently, Verboeket *et al*. reported that factors other than HIV may, both in HIV-positive and negative men who have sex with men, contribute to a low CD4+/CD8+ ratio and high CD8+ cells count^29^. According to their analysis, CMV-infection could explain this observation. In our study, the highest prevalence of CMV-infected patients was observed in the pHIVy group; however, further investigations about the effects of CMV-infection on immune system are necessary.

Few studies about TL were performed, mostly including HIV-infected adults. In HIV-infected adults naïve to cART, CD4+/CD8+ ratio was significantly associated with shorter TL. However, this association disappeared when the model was adjusted for HIV viral load^30^. No significant association was observed between cART duration and TL after adjusting for age and markers of disease progression in a group of HIV-positive adults on efficient cART^31^. Moreover, in perinatally infected children an uncontrolled HIV viremia rather than cART exposure was associated with acceleration of blood telomere attrition^32^. To our knowledge, this is the first study in which immunosenescence parameters, clinical and epidemiological variables were studied and compared between pHIVy (for long periods on efficient cART) and npHIVy.

We acknowledge some limitations in our study, including the small single-center cohort design and the only inclusion of aviremic patients. Secondly, nor CD4+ T-cell nadir neither viremia (copy-years) were available for pHIVy group. Moreover, data on separated CD4+ or CD8+ cell subsets were not collected. Despite these limitations, strengths of our investigation include the study of the impact of clinical, virological and therapeutic characteristics on immunological aging comparing pHIVy and npHIVy.

## Conclusions

To sum up, pHIVy on durable successful cART, despite long HIV duration, previous AIDS events or cART-free periods, have immune patterns similar to those of coetaneous patients infected with HIV during early adulthood, despite showing higher percentage of CD8+ cells and lower CD4+ CD8+ ratio. Moreover, CD4+ is the only variable independently associated with thymic function. Our data suggest that a good level of thymic activity can compensate the deleterious effects of past periods without cART, if HIV replication is suppressed for a sufficient time.
